# NMR-based Metabolomics and Fatty Acid Profiles to Unravel Biomarkers in Preclinical Animal Models of Compulsive Behavior

**DOI:** 10.1021/acs.jproteome.1c00857

**Published:** 2022-02-10

**Authors:** Ana C. Abreu, Santiago Mora, Ana Isabel Tristán, Elena Martín-González, Ángeles Prados-Pardo, Margarita Moreno, Ignacio Fernández

**Affiliations:** † Department of Chemistry and Physics, Research Centre CIAIMBITAL, University of Almería, Ctra. Sacramento, s/n, 04120 Almería, Spain; ‡ Department of Psychology and Health Research Center CEINSA, University of Almería, Ctra. Sacramento, s/n, 04120 Almería, Spain

**Keywords:** compulsive behavior, schedule-induced polydipsia, biomarkers, NMR, metabolomics

## Abstract

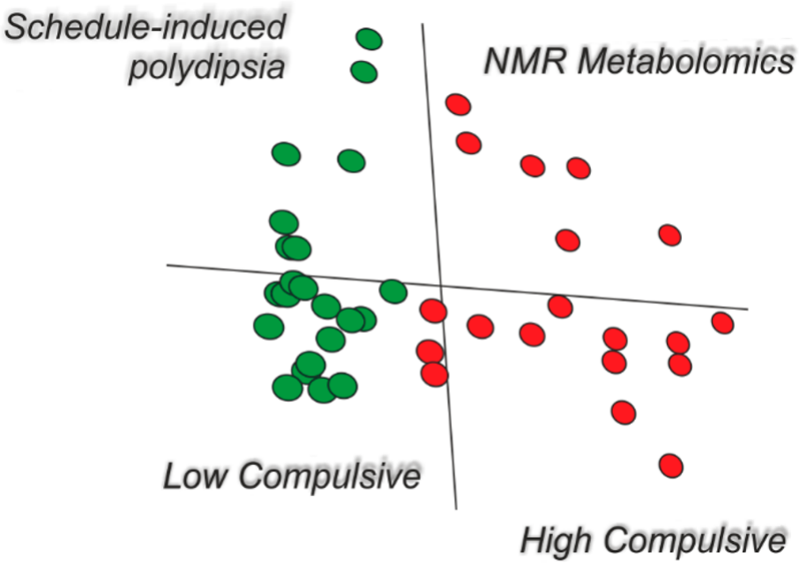

Compulsivity is a key manifestation of inhibitory control deficit and a cardinal symptom of psychopathological conditions such as obsessive-compulsive and attention-deficit hyperactivity disorders, in which metabolic alterations have raised attention as putative biomarkers for early identification. The present study assessed the metabolic profile in a preclinical model of a compulsive phenotype of rats. We used the schedule-induced polydipsia (SIP) method to classify male Wistar rats into high drinkers (HDs) or low drinkers (LDs) according to their compulsive drinking rate developed by exposure to a fixed-time 60 s (FT-60) schedule of reinforcement with water available *ad libitum* during 20 sessions. Before and after SIP, blood samples were collected for subsequent serum analysis by nuclear magnetic resonance spectroscopy coupled to multivariate analysis. Although no differences existed in the pre-SIP set, the compulsive drinking behavior induced remarkable metabolic alterations: HD rats selected by SIP exhibited a hyperlipidemic, hypoglycemic, and hyperglutaminergic profile compared with their low-compulsive counterparts. Interestingly, these alterations were not attributable to the mere exposure to reward pellets because a control experiment did not show differences between HDs and LDs after 20 sessions of pellet consumption without intermittent reinforcement. Our results shed light toward the implication of dietary and metabolic factors underpinning the vulnerability to compulsive behaviors.

## 1 Introduction

Compulsivity is defined as a perseveration of a response that is irresistible, inappropriate, and unavoidable despite its negative consequences.^1^ It is the core feature observed in obsessive-compulsive disorder (OCD), although it is present in other neuropsychopathological conditions such as schizophrenia, autism, attention-deficit hyperactivity disorder, and addiction.^2−4^ These are considered as impulsive compulsive spectrum disorders, with a high prevalence (1–3%) in Western countries and an approximate economic cost of $5 billion per year according to the World Health Organization.^5^ However, little is known about the relation between metabolic factors and inhibitory control deficit. Research on the possible identification of metabolic biomarkers underlying inhibitory control deficit and the effect of diet on the nervous system and behavior could help scientists and physicians to improve their knowledge about new mechanisms for prevention and treatment in psychopathological disorders.

Metabolomics has been widely applied in biomedicine to provide a precise analysis of small molecules (<1500 Da) associated with human metabolism. Unlike DNA, RNA, or proteins, metabolites can accurately reflect the most direct metabolic changes in our body under a certain condition in a short time period and are thus good indicators of the onset and progression of human diseases.^6^ So, the analysis of metabolites represents a sensitive measure of biological status in health or under disease.^7^ Nuclear magnetic resonance (NMR) spectroscopy offers the unique prospect to holistically screen several metabolites with a non-*a*-*priori* selection in diverse matrices as biological fluids and even in tissue biopsies.^8^ NMR-based metabolomics analysis coupled to multivariate statistical techniques have been incorporated into agricultural culture programs and clinical disease research to identify unique metabolite biomarkers in the quest for nutritional or organoleptic advantages or in a specific human disease, respectively. When the focus is placed on the identification of disease-associated biomarkers, this could allow us to (1) predict and diagnose diseases and their stages, (2) provide insights into underlying pathways in the pathogenesis and progression of the diseases, and (3) aid in disease treatment by assessing the efficacy and mechanism of action of therapeutic solutions.^6,9^

The use of animal models with higher translational power, associated with neurobehavioral endophenotypes based on biomarkers, has enabled us to bridge the gap between (dys)functional neuronal circuits and psychological constructs with bigger predictive strength than the current psychiatric nosology.^10^ However, to the best of our knowledge, only a few studies have investigated metabolomic biomarkers in animal models. In a recent study, Perez-Fernandez et al.^11^ found a hyperlipidemic and hypoglycemic profile in animals exposed to Chlorpyrifos, which also showed behavioral alterations, that is, impairments in the reaction to social novelty in the Crawley social test.

Schedule-induced polydipsia (SIP) is one of the most well-established preclinical models for the study of neuropsychopathological disorders presenting compulsive behavior, such as OCD, schizophrenia, and alcohol abuse. (For a review, see Moreno and Flores).^12^ The SIP procedure is characterized by the development of an adjunctive behavior of excessive and persistent drinking, which is nonregulatory and does not rely on physiological demands, in food-deprived animals exposed to intermittent food-reinforcement schedules in operant chambers with water available *ad libitum*.^13,14^ However, there are important individual differences in the development of adjunctive drinking after 15–20 sessions in SIP. Rats can be separated into two groups according to their rates of drinking, one with high or compulsive drinking (HD rats) and a second group with low drinking or no SIP acquisition (LD rats). This phenomenon resembles the key features of human compulsivity and OCD, thus allowing us to identify vulnerable populations based on their endophenotype (for a review, see Moreno and Flores)^12^ and entailing an outstanding chance of studying compulsive behavior under laboratory conditions.

In this sense, the promising role of metabolism as a putative contributing factor concerning vulnerability to inhibitory control deficit necessitates further research; specifically, the use of biomarkers for the early identification of such conditions creates the context for a better understanding of the problem and for early detection and, in the future, early intervention. The present study aims to add evidence in that direction with the aid of SIP.

## 2 Materials and Methods

### 2.1 Subjects

Forty male Wistar rats (Envigo, Spain) were used in this study (40 for the SIP experiment, 20 of them for the control experiment) and arrived at the laboratory weighing 200–250 g. They were housed in a four rats per cage (50 × 35 × 20 cm) distribution at a temperature of 22 ± 1 °C with a 12:12 h light–dark cycle with lights off at 08:00 h. They also had environmental enrichment consisting of PVC pipe tubes and wooden blocks and food and water provided *ad libitum*. Before SIP, the animals’ bodyweights were gradually reduced to 85% of their free-feeding baseline level through controlled feeding and daily weighing and then maintained throughout the experiment. Food, consisting of lab chow, was provided daily ∼30 min after each experimental session. All of the testing occurred between 9:00 am and 2:00 pm. All procedures were in accordance with the Spanish Royal Decree 53/2013 on the protection of experimental animals and the European Directive 2010/63/EU and approved by the Animal Research Committee of the University of Almería. We declare that the research shows commitment to the 3Rs principle (replacement, reduction, refinement).

### 2.2 Experimental Design

**Figure 1:**
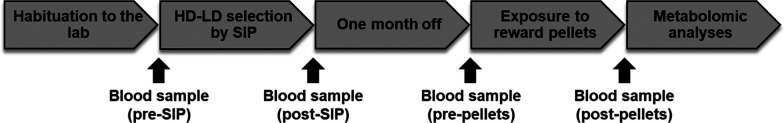
Experimental procedure illustrated in a timeline. After habituation to the lab, animals were divided into high drinkers (HDs) and low drinkers (LDs) by schedule-induced polydipsia (SIP) before (pre-SIP) and after (post-SIP) blood samples were taken. After SIP, animals remained undisturbed for 1 month. Then, an additional experiment regarding exposure to reward pellets took place to assess its dietary impact on metabolomic analyses; again, blood samples were collected before (pre-pellet) and after (post-pellet) exposure.

Once all animals reached 85% bodyweight compared with their baseline, blood samples (pre-SIP) were taken (see as follows) on the day before the start of the SIP procedure. Then, after SIP, post-SIP samples were collected. After 1 month of washout (based on previous publications; see Mora et al.),^15^ half of the animals were selected for a control test concerning the dietary impact on their metabolism. Blood samples were collected before (pre-pellet) and after (post-pellet) exposure to the test pellets with water available *ad libitum*. Figure 1 shows the experimental timeline of relevant events.

### 2.3 Blood Sampling

Animals were anesthetized using isoflurane, and blood samples (1 mL) were collected from the lateral tail vein in 1.5 mL autocued plastic tubes between 9:00 am and 12:00 pm (dark cycle). The samples were allowed to stand for 10 min before centrifuging (Sigma 3-18KS, Germany) at 3000 rpm (800*g*) for 10 min at 23 °C, after which serum was collected into duplicate 0.5 mL autocued plastic tubes and stored at −80 °C until assay.

### 2.4 SIP Procedure

Rats were tested in 12 operant SIP chambers (32 × 25 × 34 cm) (MED Associates, St. Albans, VT). A previous description of the apparatus can be found in Moreno et al.^16^ Programming and data recording were performed with the aid of a computer and commercial software Med PC (Cibertec SA, Spain). Prior to SIP, two baseline water ingestion tests on successive days were performed, where the amount (in mL) of water consumed by each animal during a period of time of 60 min with free access to 60 pellets (Noyes 45 mg dustless reward pellets; TSE Systems, Germany) was measured. After 1 day of habituation to the chambers session, rats underwent 60 min daily sessions of a fixed-time 60 s (FT-60s) schedule of food pellet delivery, where bottles containing freshwater *ad libitum* were placed in the wall opposite to the pellet dispenser. Measures recorded were: (1) total amount of water (in mL) consumed, (2) total number of licks to the bottle, and (3) total number of food magazine entries. After 19 daily sessions of SIP acquisition, animals were selected in two groups, high and low drinkers (HDs and LDs, *n* = 20 in each group), according to if their drinking rates during SIP (average of water intake on the last five sessions) were above or below the group median, respectively.

### 2.5 Exposure to Diet Pellets

Half of the animals of each group (HD and LD, *n* = 10 in each group) were exposed to 19 consecutive exposure sessions similar to SIP to further assess any dietary impact of the reward pellets. The animals received the same number of pellets as during the SIP procedure (60 pellets), but in this case, the pellets were presented under mass feeding conditions without a food-reinforcement time schedule; water was available *ad libitum*. As in SIP, the consumption of all pellets was assured by the experimenters after each session.

### 2.6 Sample Preparation for NMR

For NMR experiments, 150 μL of rat blood serum was mixed with 350 μL of D_2_O containing 0.9% NaCl and the sodium salt of 3-(trimethylsilyl)propionic-2,2,3,3-*d*_4_ acid (TSP) at 0.01% (w/w). The resulting mixture was vortexed and centrifuged for 5 min at 13 500 rpm, and 500 μL of supernatants was transferred into oven-dried 5 mm NMR tubes.

### 2.7 NMR Experiments

Acquisition of ^1^H NMR spectra of serum samples was conducted as described by Perez-Fernandez et al.^11^ with some modifications. Measurements were carried out on a Bruker Avance III 600 spectrometer operating at 600.13 MHz, equipped with a 5 mm QCI quadruple resonance pulse field gradient cryoprobe and a SampleJet autosampler, at 293 ± 0.1 K and without rotation. The water-suppressed Carr–Purcell–Meibom–Gill (CPMG) pulse sequence was applied with a total spin echo delay of 100 ms (τ – 180° – τ, 400 μs – 37 μs – 400 μs) to attenuate broad signals from protein signals. The spectrometer transmitter was locked to D_2_O frequency. Acquisition parameters were set as follows: NS = 60, DS = 16, size of fid = 32K, spectral width = 22.0 ppm, acquisition time = 1.24 s, relaxation delay = 3 s, number of loops = 120, line broadening = 0.3 Hz, receiver gain = 203. Spectra were automatically phased, baseline-corrected, and calibrated to TSP signal at 0.0 ppm. Acquisition and processing of NMR spectra were carried out by the TOPSPIN software (version 3.6.2). Metabolite assignments were performed thanks to information on scalar couplings extracted from ^1^H–^1^H COSY, ^1^H–^1^H TOCSY, ^1^H–^13^C HSQC, and ^1^H–^13^C HMBC spectra, which were recorded using standard Bruker sequences, and with the help of the Chenomx database (Chenomx, Edmonton, Canada), public NMR databases (HMDB), and literature.^17−19^ Quantification of metabolites was achieved through the integrated values of the related peak areas of nonoverlapped signals in relation to the inner standard (TSP).

### 2.8 Quantification of Fatty Acids

After NMR acquisition, serum samples were freeze-dried for 72 h. Then, the fatty acid content and profile in serum samples and also in the pellets were determined by gas chromatography (Agilent Technologies 6890 N Series Gas Chromatograph, Santa Clara, CA) after direct transesterification, as described by Rodríguez-Ruiz et al.^20^

### 2.9 Statistical Data Analysis

SIP acquisition data were analyzed using two-way repeated-measures analysis of variance (ANOVA) with between-subject factor (group: HD and LD) and within-subject factor (session: 19 sessions). *Post hoc* comparisons were performed using the Bonferroni correction. The statistical significance was set at *p* < 0.05, and the effect size was reported when appropriate: Partial η^2^ values are reported and considered as small (0.01), medium (0.06), or large (0.14) following Cohen^21^ recommendations. All analyses were carried out using Statistica software (Statsoft, version 6.0).

With respect to chemometrics analyses of ^1^H NMR spectral data, AMIX 3.9.15 (Bruker BioSpin) software was used for bucketing NMR spectra using two types of bucketing processes: (1) regular bucketing employing a bucket size of 0.04 ppm and (2) variable bucketing of NMR peaks assigned to specific metabolites (for univariate statistical analyses). In both cases, normalization was achieved by scaling the intensity of individual peaks to the total intensity recorded in the region from δ_H_ 0.2 to 10.0 ppm, except for the region of δ_H_ 5.2 to 4.74 ppm containing the residual signal of H_2_O, which was removed. NMR regular bucketed data was investigated by means of principal component analysis (PCA) in exploratory studies and by partial least-squares discriminant analysis (PLS-DA) or orthogonal partial least-squares discriminant analysis (OPLS-DA) to determine the existence of differences between experimental groups and to identify the metabolic features responsible for their discrimination. This multivariate data analysis was performed using SIMCA-P software (v. 17.0, Umetrics). The results of the cross-validation for PLS-DA and OPLS-DA models are given by means of cumulative *R*^2^ and *Q*^2^ values, where *Q*^2^ values of >0.5 were considered indicative of a good predictive model. Models were also validated by being subsequently subjected to permutation tests (a total of 100), and the new coefficients *R*^2^ and *Q*^2^ generated from the permutation test were compared with those from the real model. If intercept *R*^2^ and *Q*^2^ values from the permutation test were significantly smaller than *Q*^2^ of the real model, then the model was regarded as predictable. The statistical significance of the estimated predictive power of PLS-DA and OPLS-DA models was further assessed with an ANOVA test of the cross-validated residuals (cv-ANOVA). Models with *p* values of <0.05 were considered to have a good prediction.

Important loadings (spectral regions) for the discrimination observed from the predictive models were selected by generating the variable importance in projection (VIP) plot. Loadings with VIP scores of >1 were considered relevant to the generated PLS-DA and OPLS-DA models. ANOVA (analysis of variance) analyses followed by least significant difference (LSD) *post hoc* tests were employed to determine the significance of differences for the metabolite ratios between groups; *p* values of <0.05 were considered statistically significant. Finally, metabolic changes with false discovery rate (FDR)-adjusted *p* values (*q* values) of <0.05 were considered.

## 3 Results and Discussion

### 3.1 SIP Acquisition

**Figure 2:**
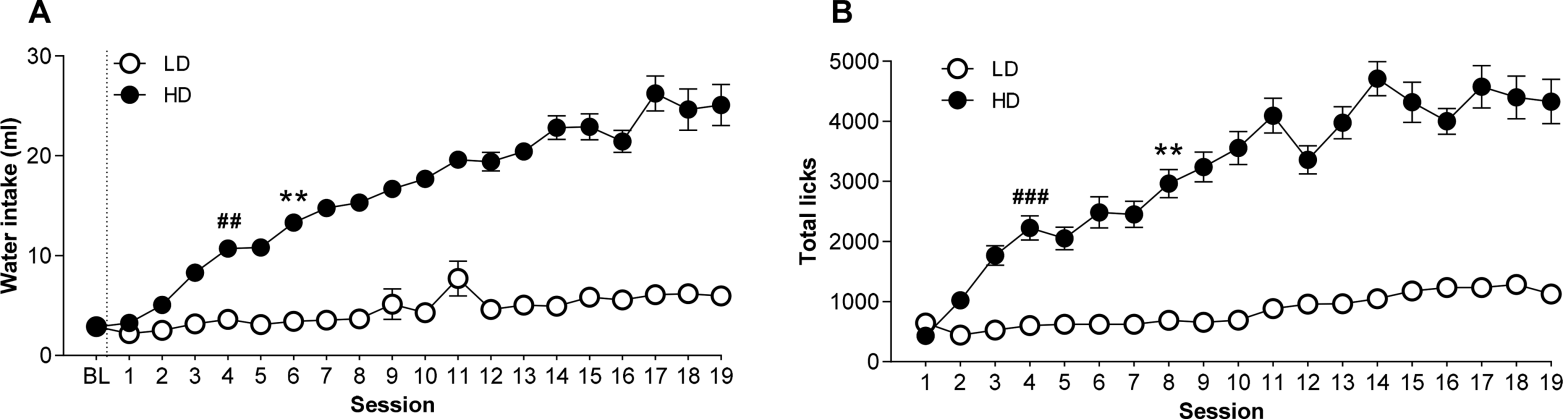
Means (±SEM) for (A) water intake and (B) number of licks in FT-60s across 19 sessions of SIP. Statistical analyses indicated significant differences between low drinkers (LDs, *n* = 20) and high drinkers (HDs, *n* = 20; ** *p* < 0.01) in both water intake and total licks. Significant differences from session 1 were found in HDs from session 6 in water intake (## *p* < 0.01) and from session 8 (### *p* < 0.001) onward.

LD and HD behavior is clearly evidenced by not only the water intake but also the number of licks, as previously mentioned. Figure 2 shows SIP acquisition and maintenance during 19 sessions. One-way repeated ANOVA measures revealed differences in the SIP acquisition concerning water intake, as shown by the interaction between sessions and group (interaction SIP session × group effect: (F18, 666 = 9.146, *p* < 0.001, partial η^2^ = 0.198). This effect was also confirmed by the significant interaction in total licks (interaction SIP session × group effect: F18, 576 = 8.445, *p* < 0.001, partial η^2^ = 0.208).

A *post hoc* comparison revealed that SIP induced different drinking behaviors across the 19 sessions in high and low drinkers: LD and HD animals exhibited remarkable differences in water intake from session 6 (*p* < 0.01) onward. Moreover, when compared with session 1, the HD group significantly increased its water consumption from session 4 (*p* < 0.01) onward. A similar pattern was found concerning total licks, where LD and HD groups differed from session 8 (*p* < 0.01) onward, and HD showed an increased number of licks from session 4 (*p* < 0.001) onward compared with session 1. No significant differences were found between LD and HD animals concerning total magazine entries.

### 3.2 Assignment of Metabolites in Blood Serum Detected by ^1^H NMR Spectroscopy

**Figure 3:**
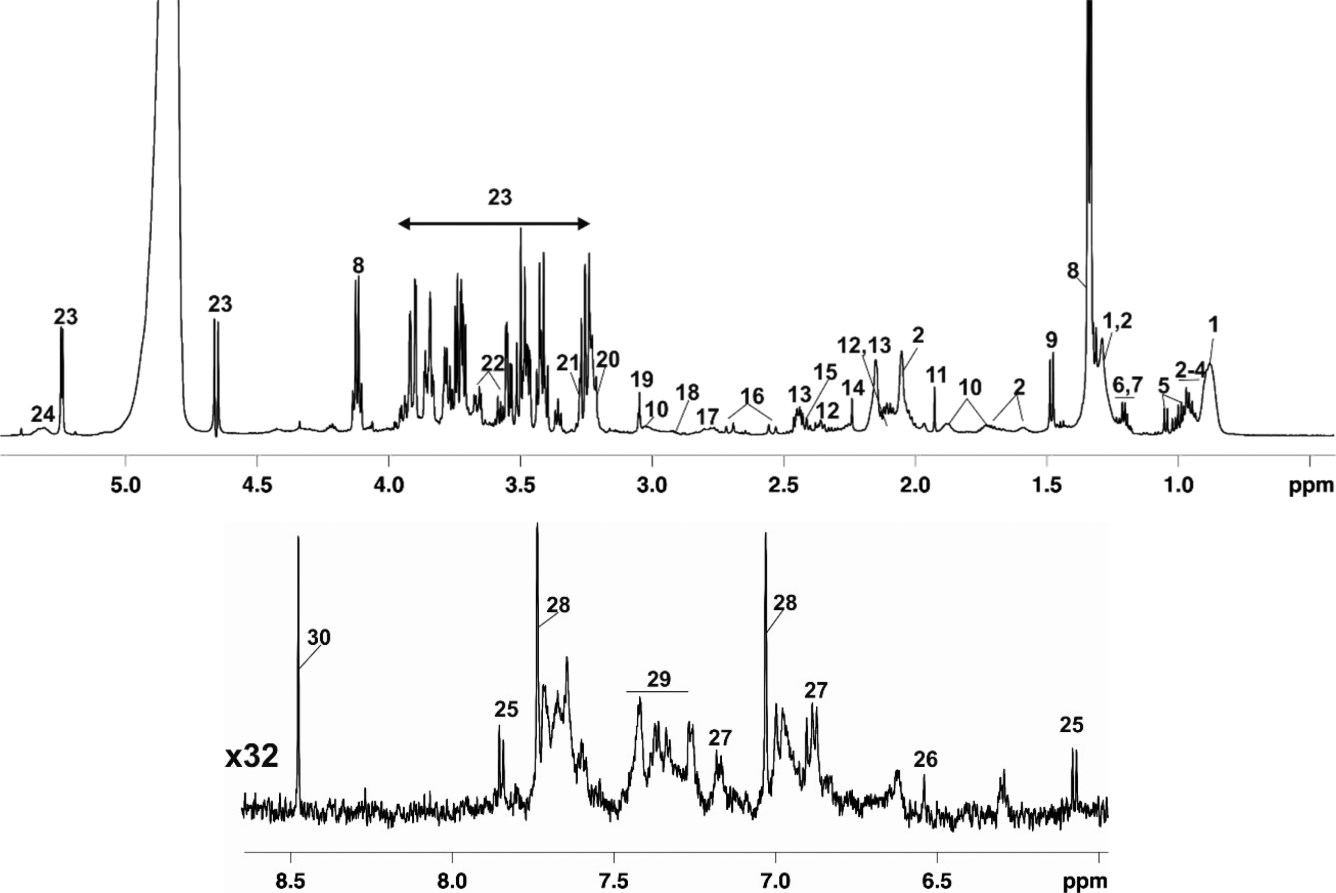
Subregions of a typical ^1^H NMR spectrum (600 MHz) of a blood serum sample taken to a rat from pre-SIP group. Numeration: **1**: LDL/VLDL, **2**: fatty acids (FA), **3**: leucine, **4**: isoleucine, **5**: valine, **6**: 3-hydroxybutyrate (3HB), **7**: EtOH, **8**: lactate, **9**: alanine, **10**: lysine, **11**: acetate, **12**: glutamate, **13**: glutamine, **14**: acetoacetate, **15**: pyruvate, **16**: citrate, **17**: PUFA, **18**: aspartate, **19**: creatine, **20**: choline, **21**: trimethylamine *N*-oxide (TMAO), **22**: glycerol, **23**: glucose, **24**: UFA, **25**: pyrimidine, **26**: fumarate, **27**: tyrosine, **28**: histidine, **29**: phenylalanine, **30**: formate.

The assignment of each metabolite present in serum samples was achieved and is illustrated in Figure 3. Table S1 provides full information on chemical shifts, multiplicity, and coupling constants for each metabolite or for each metabolite-type compound.

The assigned metabolites belong mostly to the classes of amino acids (valine, isoleucine, leucine, alanine, lysine, glutamate, glutamine, aspartate, tyrosine, and phenylalanine) and derivatives (e.g., 3-hydroxybutyrate, creatine), organic acids (acetic acid, acetoacetic acid, citric acid, and pyruvic acid), carbohydrates (α- and β-glucose), choline (Cho)-based compounds, which are essential components of cellular membranes, polyols (e.g., glycerol), and fatty acids. Trimethylamine *N*-oxide is an osmolyte used by the body to counteract the effects of increased urea concentration that accumulates during kidney failure and was also found in the spectra. Ketone bodies like 3-hydroxybutyrate, acetate, and acetoacetate, generally induced by fasting,^17^ could also be found in rat serum samples.

### 3.3 Chemometrics Analyses of ^1^H NMR Spectral Data

Multiparametric statistical tools were applied to NMR data in the analyses of rats serum samples to evaluate changes in the metabolic profiles in the presence of OCD. Blood samples were taken at four different times during the experiment: pre- and post-SIP and pre- and post-pellet (as indicated in Figure 1). An exploratory analysis of NMR data was first achieved by means of PCA that clustered similar samples together based on the input data (Figure S1). PCA is useful for revealing the major trends in the ^1^H NMR data and the possible analytical and biological confounder variables. On the basis of the PCA results, a slight division of samples between pre- and post-SIP groups (Figure S1a) and between pre- and post-pellet groups (Figure S1b) is evident, independent of the obsessive-compulsive behavior of the rats.

#### 3.3.1 Differentiation of Pre-SIP and Post-SIP Serum Samples

**Figure 4:**
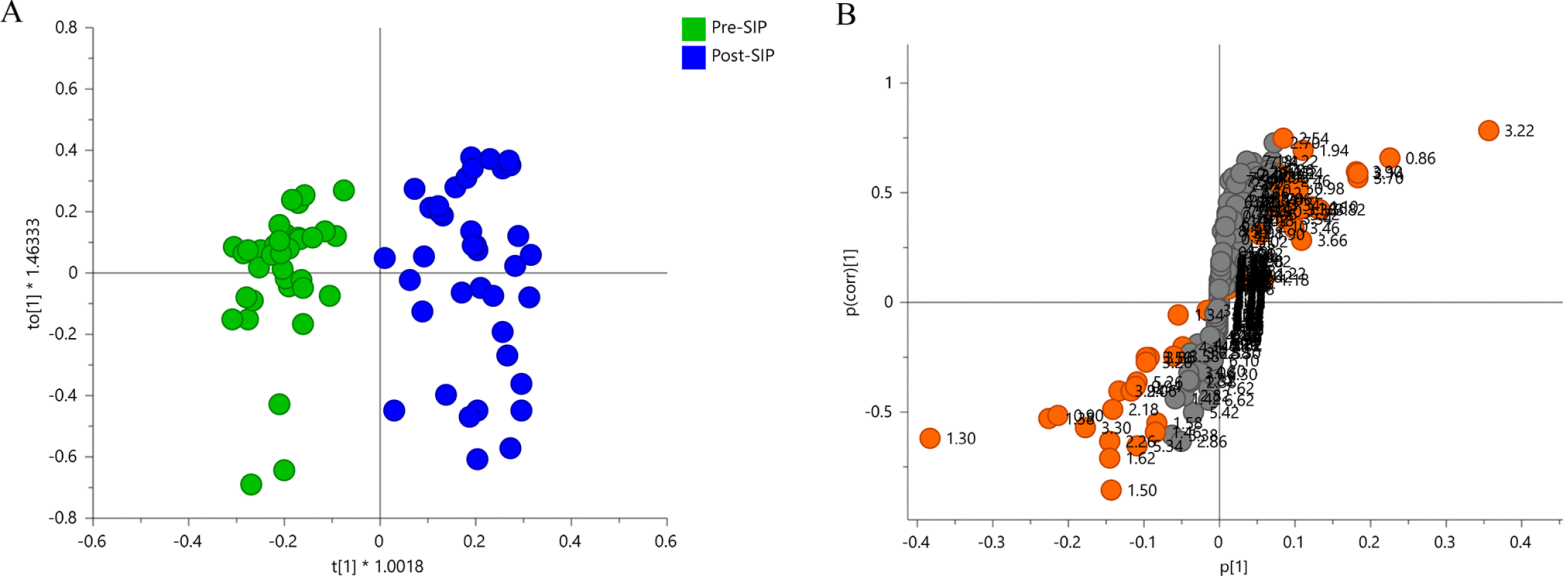
(A) OPLS-DA score plot obtained for ^1^H NMR data of samples taken prior to (*n* = 40) and after SIP (*n* = 38). (B) S-plot showing the most significant metabolites for discrimination (metabolites with VIP > 1 values are colored): Bottom left metabolites were significantly decreased (alanine: loadings 1.46, 1.50; lactate: loadings 1.34, 4.14; UFA: loadings 5.34, 2.06, 2.18, 2.26, 0.94), whereas those located at the top right were increased post-SIP (choline: loading 3.22; citrate: loadings 2.54, 2.70; acetate: loading 1.94; acetoacetate: loading 2.22; glucose: loadings 3.34, 3.38, 3.42, 3.46, 3.54, 3.66, 3.78, 3.82, 5.26; ethanol: loadings 1.18, 3.66; and LDL/VLDL/saturated fatty acids: loadings 0.86, 1.26, 1.30, 2.14). Pareto scaling was carried out. *R*^2^*X* = 0.639, *Q*^2^ = 0.764, *p*(cv-ANOVA) = 7.0 × 10^–18^. The model was validated by a permutation test (with 100 permutations).

To improve the discrimination observed between pre-SIP and post-SIP data, we applied an OPLS-DA model to the ^1^H NMR data (Figure 4A). The chosen preprocessing method contains the orthogonal signal correction (OSC), which allows one to eliminate unnecessary information. In the OSC procedure, the X matrix was corrected by a subtraction of variation orthogonal to the *y* (containing the classes for each sample, in this case, pre-SIP versus post-SIP) vector calibration. The corresponding S-plot (Figure 4B) revealed the most relevant metabolites for the discrimination (with VIP values >1). Metabolites of the bottom left were significantly decreased (alanine, lactate, unsaturated fatty acids), whereas those located at the top right were increased post-SIP (choline, citrate, acetate, acetoacetate, ethanol, glucose, and LDL/VLDL/saturated fatty acids).

The results indicate that SIP induced changes in lipid-metabolism-related molecules (fatty acids and cholesterol levels, choline, acetate, acetoacetate, glutamine) and energy metabolism (related to the tricarboxylic acid (TCA) cycle). Acetate is formed in the body by the metabolism of certain substances, in particular, in the liver in the oxidation of lipids. It is a precursor of acetyl-CoA, which is used by cells for the synthesis of fatty acids and cholesterol. To further investigate if a differential effect is induced by the paradigm in HD and LD groups, we added this variable to the model in the following analysis (detailed in Section 3.3.2).

#### 3.3.2 Differentiation between LD and HD Groups in Pre- and Post-SIP Serum Samples

**Figure 5:**
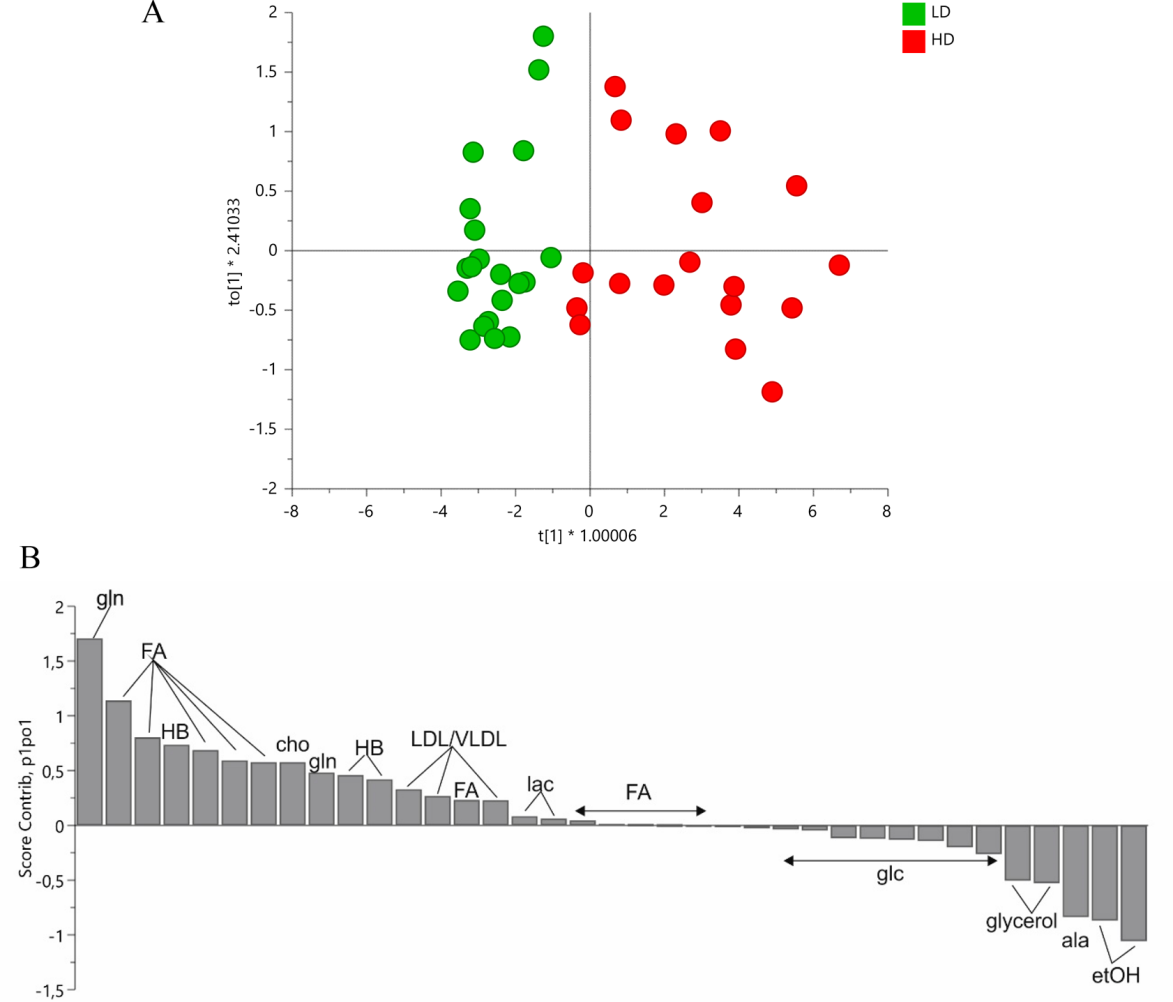
OPLS-DA (A) score and (B) contribution plots obtained for ^1^H NMR data of post-SIP samples that were classified as LD (*n* = 20) and HD (*n* = 18). Positive and negative bars in the contribution plot explain the spectral regions containing metabolites that discriminate for HD and LD samples, respectively. These include glutamine (loadings 2.14, 2.46), fatty acids/LDL/VLDL (loadings 0.86, 0.90, 0.94, 1.26, 1.30, 1.34, 2.02, 2.06, 2.26, 2.30), 3-hydroxybutyrate (HB, loadings 2.32, 2.40, 4.16), choline (loading 3.22), and lactate (1.34, 4.10), which increased in HD drinkers, and ethanol (loadings 1.18, 3.66), alanine (loading 1.50), glycerol (loadings 3.62, 3.66), and glucose (loadings 5.26, 4.66, 3.90, 3.86, 3.74, 3.46, 3.26), which decreased. Only the samples from very low drinkers (<10 mL) and very high drinkers (>20 mL) were considered. Pareto scaling was carried out. *R*^2^*X* = 0.68, *Q*^2^ = 0.58, *p*(cv-ANOVA) = 0.032.

A valid discrimination between LD and HD drinkers was found only for post-SIP samples after the application of an OPLS-DA model (Figure 5). This means that it was not possible to observe metabolic changes *a priori* to the SIP procedure, so it would not be possible to “predict” the behavior of each rat based on serum metabolic profiles. To obtain a valid model, we removed serum samples of rats showing a drinking volume between 10 and 20 mL from the model. So, this model tried to maximize differences between very low drinkers (<10 mL) and very high drinkers (>20 mL).

**Figure 6:**
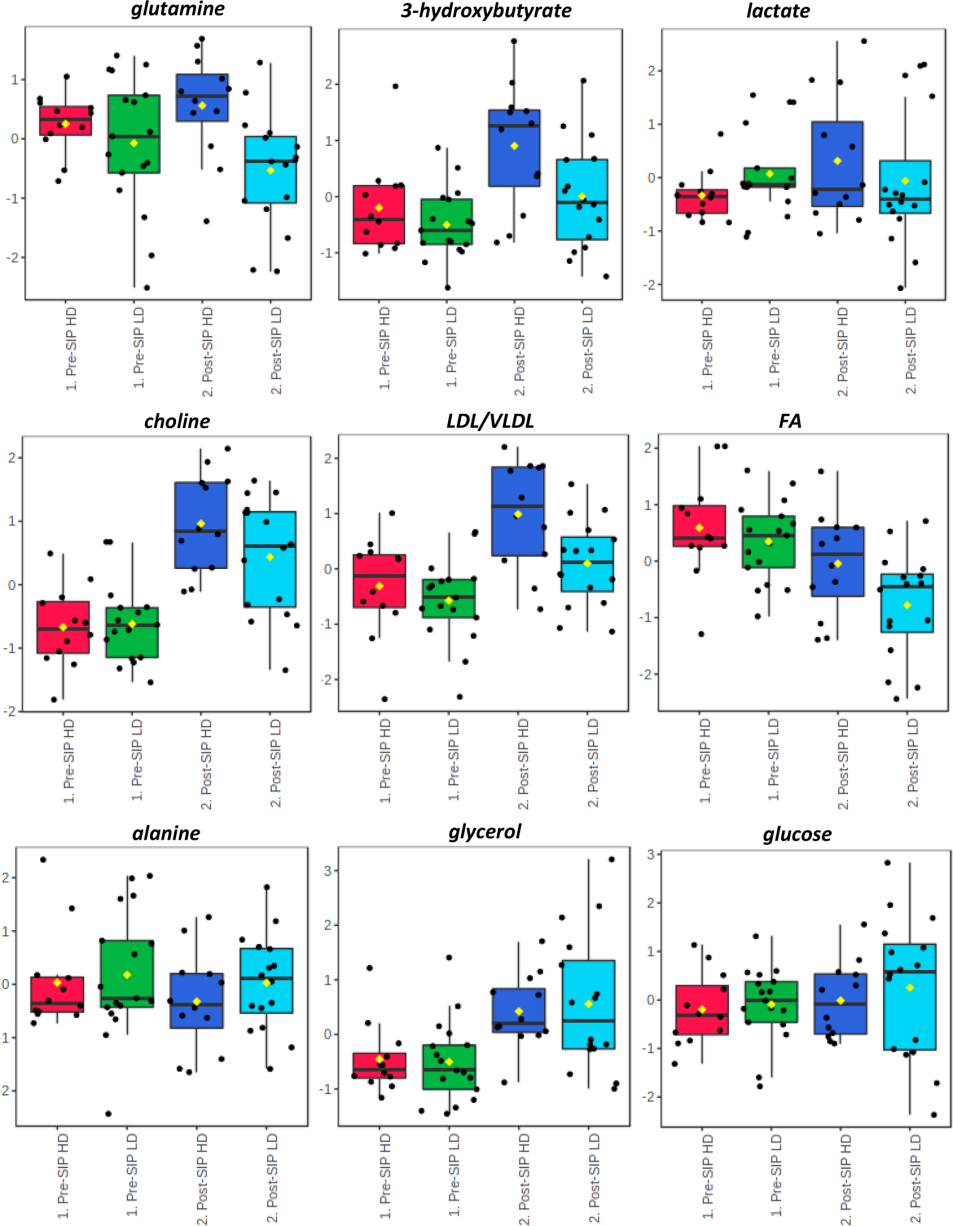
Box plots showing the average, median quartiles, and extremes for specific peak integration values (relative to the TSP peak integral and normalized to total spectra intensity) for 3-hydroxybutyrate, glutamine, lactate, choline, LDL/VLDL, and FA levels, except for UFA, which increased post-SIP for HD rats, and glycerol, alanine, and glucose, which decreased post-SIP for HD rats when compared with LD rats. All of these metabolites presented *p* values of <0.05 using ANOVA analyses followed by the least significant difference (LSD) *post hoc* tests in post-SIP.

The blood serum of post-SIP HD rats specifically showed increased low-density lipoprotein (LDL)/very low-density lipoprotein (VLDL), fatty acid (except unsaturated fatty acids), 3-hydroxybutyrate, glutamine, choline, and lactate levels, accompanied by a decrease in glycerol, glucose, alanine, and ethanol levels when compared with post-SIP LD rats. Figure 6 shows the box-and-whisker plots of normalized integration values of peaks from these altered metabolites (relative to TSP signal integral) with an indication of the integration ranges, median quartiles, and extremes.

**Figure 7:**
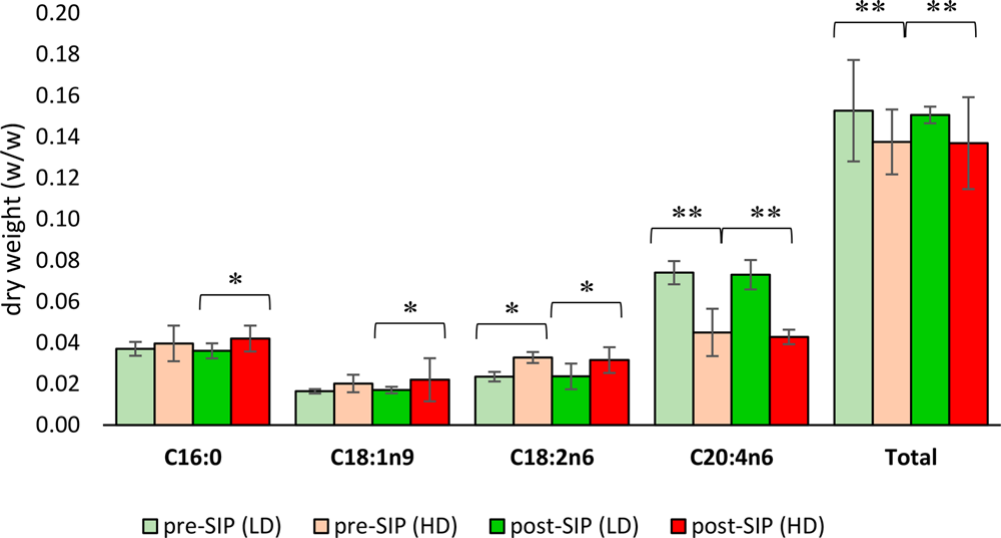
Fatty acid quantification by GC-FID. Bars with (*) and (**) revealed a significant increase and decrease (*p* < 0.05 by univariate *t* tests), respectively, in the lipid content in the serum of HD rats compared with LD rats.

These results suggest that lipid-metabolism-related molecules (including total cholesterol (TC) content, glutamine, choline, and glycerol) might be associated with a compulsive behavior phenotype during SIP. To further support the observed trends, we conducted the identification and quantification of fatty acid profiles in serum samples by gas chromatography with flame ionization detection (GC-FID) (Figure 7). The polyunsaturated arachidonic acid (C20:4n6) was found to be the major fatty acid in the samples followed by the saturated palmitic acid (C16:0) and by linoleic acid (C18:2n6) and oleic acid (C18:1n9). HD rats showed an increase in the saturated palmitic acid (C16:0), the monounsaturated oleic acid (C18:1n9), and the polyunsaturated linoleic acid (C18:2n6) compared with LD rats (*p* < 0.05) and a decrease in arachidonic acid, the major polyunsaturated fatty acid (PUFA) in serum samples. As shown in Figure 7, statistically significant pre-SIP differences between LDs and HDs exist and remain constant post-SIP. These results agree well with NMR results that detected an increase in fatty acid/LDL/VLDL content in HD rats, except for UFA. Interestingly, GC-FID analysis showed that the total PUFA content was significantly higher in LD rats compared with HD rats already in pre-SIP samples (*p* < 0.05).

These results are intriguing because some preclinical studies have also found a similar output in compulsive animals: Increased TC, VLDL, and LDL levels were found in dogs exhibiting tail chasing (a stereotyped behavior proposed as a valid preclinical model for studying OCD in animals)^22^ in comparison with control subjects,^23,24^ whereas decreased glucose serum levels were found in highly compulsive rats selected by SIP compared with noncompulsive animals.^25^ In human studies, OCD patients have been shown to exhibit higher high-density lipoprotein (HDL) serum levels than healthy controls.^26^ Moreover, Brennan et al.^27^ performed a critical review of ^1^H magnetic resonance imaging studies on OCD in brain tissues and concluded that changes in glutamate/glutamine levels were common among patients versus healthy individuals, although some inconsistencies have been unraveled. For instance, some studies reported a decrease whereas others reported an increase in the glutamate-glutamine (Glx) level. Also, choline was referred to be a crucial biomarker in OCD, and most studies showed an increase in this metabolite in OCD individuals.

Similarly, other neuropsychiatric conditions related to inhibitory control have been studied regarding metabolic changes. Schwarz et al.^28^ reported significant alterations of brain tissue free fatty acids and phosphatidylcholine levels in subjects with schizophrenia and bipolar disorder using a high-throughput mass spectrometry approach (UPLC–MS) and suggested that lipid abnormalities may be an intrinsic feature of both schizophrenia and bipolar disorder. Atmaca et al.^29^ observed decreased serum cholesterol and leptin levels in bipolar disorder patients, whereas Ozbulut et al.^30^ found decreased serum ghrelin and increased TC levels in euthymic patients under lithium treatment when compared with controls. In depression, Kaddurah-Daouk and Krishnan^31^ reported that fatty acids, glycerol, and γ-aminobutyric acid (GABA) were altered in currently depressed patients when compared with controls. Also, an increase in the concentration of the ketone 3-hydroxybutyric acid was found in remitted patients relative to depressed patients. Furthermore, Nakazato et al.^32^ found a relationship between increased glutamine serum levels and compulsive-like behavior, as assessed by total and perseverative errors in set-shifting tasks in subjects recovered from anorexia nervosa, which also is adopted in the impulsive-compulsive spectrum.

It could be argued, however, that these metabolic effects might have been induced by diet due to the mere exposure to reward pellets during SIP sessions. Indeed, obese individuals have been reported to exhibit poorer behavioral inhibition^18^ and a stronger attentional bias toward food^33,34^ compared with healthy subjects. Moreover, impulsive behavior seems to be correlated with a higher consumption of fast food^35^ and a higher body mass index (BMI),^36^ whereas in preclinical models, exposure to highly palatable, fat-rich diets has been shown to induce compulsive food-seeking behavior^37−39^ and to affect marble burying behavior.^40−42^ Thus to explore that possibility, we planned and carried out an additional experiment where a cohort of animals were subjected to 19 sessions of exposure to reward pellets, as described in the Methods section.

#### 3.3.3 Differentiation between Pre-Pellet and Post-Pellet Serum Samples

**Figure 8:**
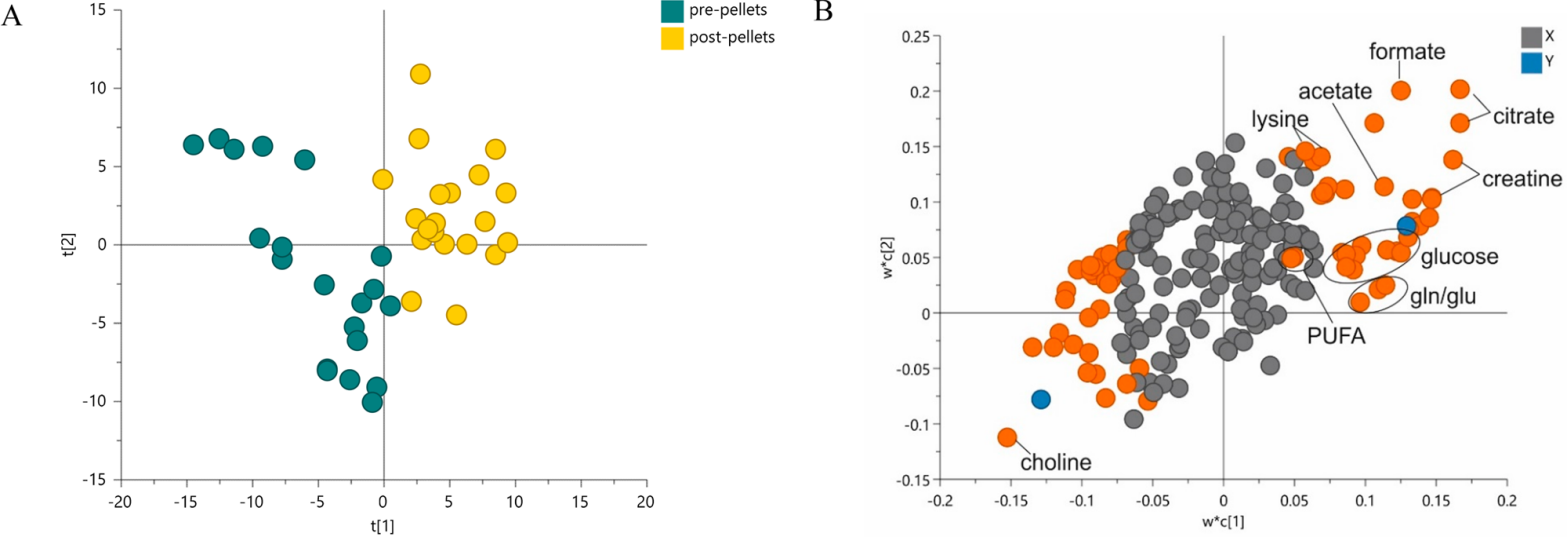
(A) PLS-DA score plot obtained for ^1^H NMR data of samples before (*n* = 20) and after (*n* = 20) changing the diet to reward pellets (pre- and post-pellet, respectively). (B) PLS-DA loading plot revealing the most significant metabolites for discrimination (with VIP values >1), which correspond to creatine (loadings 3.06, 3.94), glucose (loadings 5.26, 3.94, 3.78, 3.74, 3.56, 3.50, 3.26), PUFA (loading 2.78), lysine (loadings 1.50, 1.74), acetate (loading 1.94), formate (loading 8.46), and glutamine/glutamate (loadings 2.14, 2.46, 2.36), which increased in post-pellet samples, and choline (loadings 3.22), which decreased. Unit-variance scaling was carried out. *R*^2^*X* = 0.65, *Q*^2^ = 0.90, *p*(cv-ANOVA) = 6.5 × 10^–9^. The model was validated by a permutation test (with 100 permutations).

A PLS-DA model was generated to investigate the metabolic differences between serum samples collected before and after the control diet experiment. A valid discrimination between pre- and post-pellet groups was found in the score plot (Figure 8A), meaning that the reward pellets had effects on the serum metabolic composition. The loading plot (Figure 8B) highlights the most significant variables of the model by describing the influence and relation among the variables in the model plane. Therefore, it is possible to conclude that the peaks in spectral zones should be significantly increased (upper right loadings) and decreased (bottom left loadings) in the blood serum due to the exposure to reward pellets in this control experiment (VIP values >1) and also due to a physiological metabolic evolution over time. An increase in creatine, citrate, glucose, lysine, glutamine/glutamate, acetate, and PUFAs was detected in the blood serum after such exposure. The spectral zones in the bottom left of the loading plot referring to the metabolites that decrease in post-pellet serum include choline and other spectral regions that mostly correspond to noise and were not relevant for analyses, so these were not considered.

As in the previous set of experiments, the lipid profile of the pellets was investigated and quantified by GC-FID (Table S2). The reward pellets during the SIP experiment showed a 2.3-fold increase in fatty acid content, including on PUFAs. Because the model was not able to discriminate between HDs and LDs either pre- or post-exposure to reward pellets, it is plausible to assume that such alterations affected both groups equally, thus pointing to the idea that the SIP paradigm, and not the diet, is responsible for the metabolic alterations observed in the vulnerable subjects, the HD group. There are several underlying mechanisms that should be investigated in the future regarding the metabolic alterations observed in the compulsive-drinking HD group selected by SIP, for example, the possible role of an altered vasopressin, a hormone implicated in the metabolic syndrome, that drives fat production as a mechanism for storing metabolic water.^43^ Furthermore, the relevance of gut microbiota dysregulation in different neuropsychopathological disorders^44−46^ should be considered in the metabolic alterations observed in compulsive HD rats. In this sense, in our laboratory, we have demonstrated that compulsive HD rats showed a lower bacterial diversity than LD rats, irrespective of the diet.^47^ However, the same study also demonstrated that the administration of a tryptophan-depleted diet reduced bacterial evenness and showed a highly functionally organized community in the compulsive HD rats selected by SIP. This points toward a bacterial community that is fragile to external changes due to the dominance of a low number of species in compulsive HD rats compared with noncompulsive LD rats.

The dramatic effect found in the present work adds evidence not only to the stressful properties of intermittent reinforcement in SIP inducing compulsive behavior but also to severe physiological effects, such as increased corticosterone levels,^25^ increased amygdaloid and decreased hippocampal volume,^15^ and increased dendritic spinal density in the dorsal striatum.^48^ In this sense, the aforementioned finding of increased glutamine levels in HD in the present study is not surprising given its major role in the brain as the precursor of glutamate, which is a key factor regarding neuroplasticity^49,50^ and one of the altered mechanisms^51^ and putative therapeutic targets^52^ in compulsive drinking in SIP.

## 4 Conclusions

The present study has investigated the metabolomic profile by means of NMR of high- and low-compulsive rats selected by SIP as a potential tool for identifying critical biomarkers in vulnerable subjects. We found that although SIP itself induced a change in the metabolomic profile, it affected the HD animals differently, which showed a hyperlipidemic, hypoglycemic, and hypoglutaminergic profile, compared with the LD animals, in line with the literature regarding both preclinical models and human patients in OCD and related disorders. Moreover, mere exposure to reward pellets did not result in a valid model for predicting the phenotypic profile based on metabolomics, thus leading us to discard an effect due to diet alone. Our data add significant evidence to a crucial topic in basic neuroscience and potentially clinical fields; however, future studies are ongoing in our laboratories to unscramble this complex phenomenon and to further characterize the putative role of metabolism in the triggering and early identification of latent vulnerabilities. In addition, the putative impact of a high-fat diet on inhibitory control deficit and the potential use of metabolomic biomarkers in the early diagnose of compulsive spectrum disorders are envisaged as well.

## Supporting Information Available

Table S1. Peak assignment of metabolites identified. Table S2. GC-FID fatty acid profiles. Figure S1. PCA score plots obtained from ^1^H NMR data (PDF)
