# Supplementary material for: NMR-based Metabolomics and Fatty Acid Profiles to Unravel Biomarkers in Preclinical Animal Models of Compulsive Behavior
Source: J Proteome Res. 2022 Feb 10;21(3):612–22. doi: 10.1021/acs.jproteome.1c00857 (PMC8902800; doi:10.1021/acs.jproteome.1c00857)
Supplement: Supplementary file 1 — pr1c00857_si_001.pdf [file pr1c00857_si_001.pdf]

Supplementary Information

NMR-based metabolomics and fatty acid profiles to unravel biomarkers in preclinical animal models of compulsive behavior

Ana C. Abreu[1],† Santiago Mora[2],† Ana Isabel Tristán[1], Elena Martín-González[2], Ángeles Prados-Pardo[2], Margarita Moreno[2,]* and Ignacio Fernández[1,]*

[1] Department of Chemistry and Physics, Research Centre CIAIMBITAL, University of Almería, Ctra. Sacramento, s/n, 04120, Almería, Spain

[2] Department of Psychology and Health Research Center CEINSA, University of Almería, Ctra. Sacramento, s/n, 04120, Almería, Spain

† These authors contributed equally.

Table of Contents

Table S1. Peak assignment of metabolites identified……………………………S2-S3

Table S2. GC-FID fatty acid profiles………………………………………………….S4

Figure S1. PCA score plots………………………………………………………S5

Table S1. Peak assignment of metabolites identified on blood serum samples prepared in 0.9% NaCl (D_2O) and TSP (0.01%, ww) by NMR

Metabolite	Chemical shift (ppm) [multiplicity, coupling constants (Hz)]
1. LDL/VLDL	0.84 - 0.92 (b.s.), 1.26 – 1.36 (b.s.)
2. FA	0.87 (b.s. –C**H**$_3$, FA except *n*-3), 0.96 (b.s. –C**H**$_3$, *n*-3 FA), 1.26 – 1.36 (b.s., –(C**H**$_2$)$_n$–), 1.61 (b.s., –C**H**$_2$–CH$_2$–COOR,), 2.04 (b.s., –C**H**$_2$–CH=CH–, UFA), 2.26 (b.s.,–C**H**$_2$–COOR)
3. Leucine	0.97 (d, *J* = 6.3 Hz), 0.98 (d, *J* = 6.3 Hz)
4. Isoleucine	1.02 (d, *J* = 7.0 Hz), 0.96 (t, *J* = 7.0 Hz)
5. Valine	1.05 (d, *J* = 7.2 Hz), 1.00 (d, *J* = 7.1 Hz)
6. 3-Hydroxybutyrate	1.21 (d, *J* = 6.2 Hz), 2.30 (m), 2.39 (m), 4.17 (m)
7. Ethanol	1.20 (d, *J* = 6.9 Hz), 3.68 (q, *J* = 6.9 Hz)
8. Lactate	1.32 (d, *J* = 6.8 Hz), 4.13 (q, *J* = 6.7 Hz)
9. Alanine	1.48 (d, *J* = 7.2 Hz)
10. Lysine	1.49 (m), 1.72 (m), 1.85 (m), 3.03 (t, *J* = 7.7 Hz)
11. Acetate	1.94 (s)
12. Glutamate	2.08 (m), 2.36 (m), 3.75 (m)
13. Glutamine	2.14 (m), 2.46 (m), 3.78 (m)
14. Acetoacetate	2.25 (s)
15. Pyruvate	2.39 (s)
16. Citrate	2.55 (d, *J* = 16.2 Hz), 2.71 (d, *J* = 16.2 Hz)
17. PUFA	2.80 (m)
18. Aspartate	2.87 (dd, *J* = 16.0, 4.4 Hz), 2.74 (dd, *J* = 16.0, 8.5 Hz)
19. Creatine	3.05 (s), 3.93 (s)

20. Choline	3.22 (s)
21. Trimethylamine *N*-oxide	3.28 (s)
22. Glycerol	3.67 (dd, J = 11.4; 4.4 Hz), 3.58 (dd, J = 11.4; 6.3 Hz)
23. Glucose	5.25 (d, J = 3.6 Hz), 4.66 (d, J = 8.2 Hz)
24. UFA	5.34 (b.s.)
25. Nucleoside	6.07 (d, J = 3.4 Hz), 7.84 (d, J = 7.3 Hz)
26. Fumarate	6.54 (s)
27. Tyrosine	6.89 (d, J = 8.3 Hz), 7.18 (d, J = 8.3 Hz)
28. Histidine	7.07 (s), 7.37 (s)
29. Phenylalanine	7.36 (m), 7.32 (m), 7.25 (m)
30. Formate	8.47 (s)

* Assignment of metabolites was based on the analysis of homo- and heteronuclear 1D and 2D NMR experiments (^{1}H-NOESY, ^{1}H-^{1}H TOCSY, ^{1}H-^{1}H COSY, ^{1}H-^{13}C edited HSQC and ^{1}H-^{13}C HMBC) and on the use of the Chenomx NMR database, public NMR databases, and literature data. Abbreviations: b.s.: broad signal, m: multiplet, d: doublet, dd: doublet of doublets, s: singlet

Table S2. GC-FID fatty acid profiles (in w/w) of pellets used before (Pre-SIP) and during the SIP task. In bold is given the overall content.

**Fatty acid**	**Pre-SIP pellet**	**SIP pellet**
Octoic acid (C8:0)	0.00 ± 0.00	0.11 ± 0.00
Capric Acid (C10:0)	0.05 ± 0.00	0.07 ± 0.00
Lauric acid (C12:0)	0.00 ± 0.00	0.09 ± 0.01
Palmitic acid (C16:0)	0.53 ± 0.03	0.96 ± 0.09
Oleic acid (C18:1n9)	0.47 ± 0.02	1.46 ± 0.13
Linoleic acid (C18:2n6)	1.26 ± 0.10	2.72 ± 0.12
α-Linolenic acid (C18:3n3)	0.10 ± 0.00	0.04 ± 0.00
**TOTAL**	**2.41 ± 0.15**	**5.46 ± 0.35**

A)

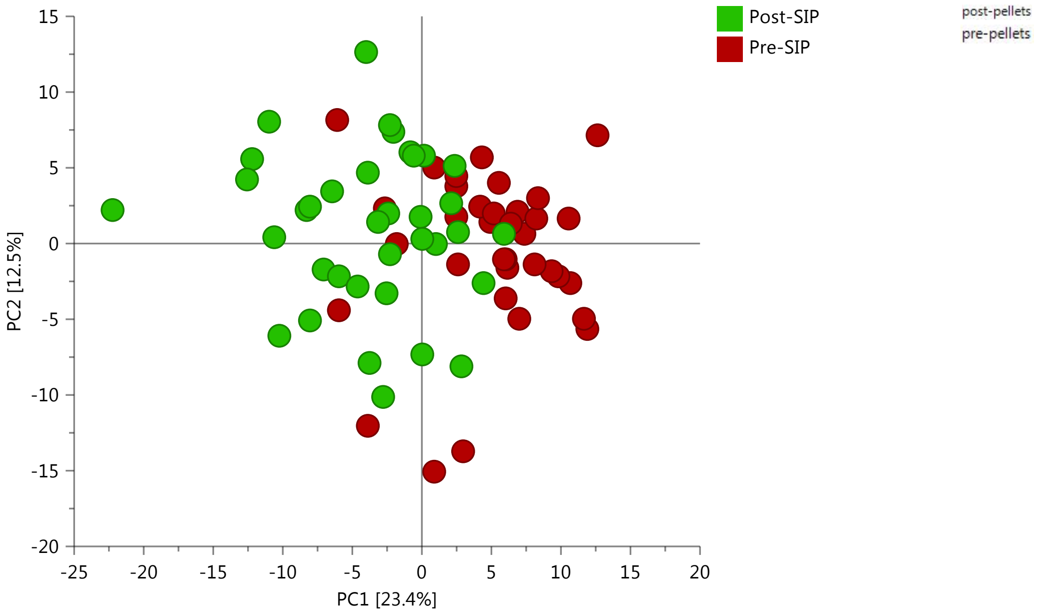

B)

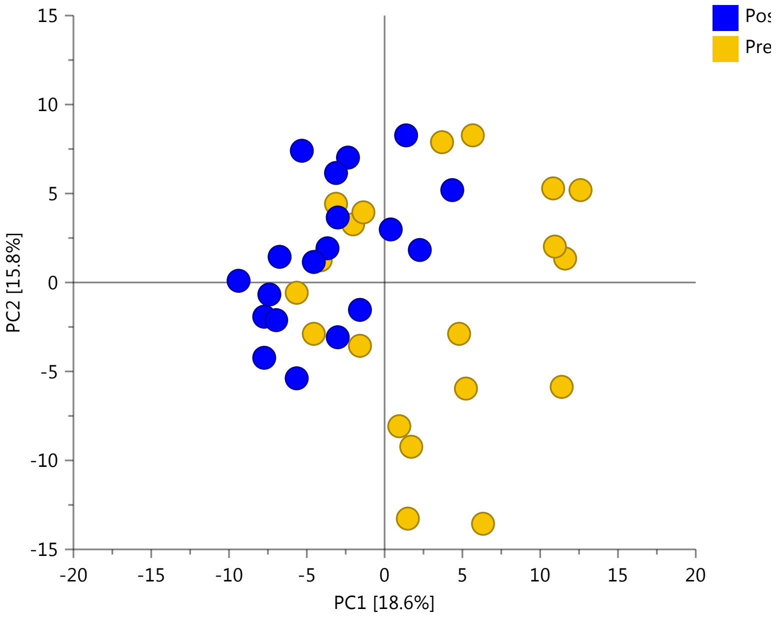

Figure S1. PCA *scores* plots (first two components) obtained for ^{1}H NMR data of **(A)** pre- and post-SIP serum samples, and **(B)** pre- and post-pellets serum samples. Scaling was done to unit variance.
